# Deubiquitination of MITF-M Regulates Melanocytes Proliferation and Apoptosis

**DOI:** 10.3389/fmolb.2021.692724

**Published:** 2021-06-09

**Authors:** Shuaishuai Hu, Shaocheng Bai, Yingying Dai, Naisu Yang, Jiali Li, Xiyu Zhang, Fan Wang, Bohao Zhao, Guolian Bao, Yang Chen, Xinsheng Wu

**Affiliations:** ^1^ College of Animal Science and Technology, Yangzhou University, Yangzhou, China; ^2^ Animal Husbandry and Veterinary Research Institute Zhejiang Academy of Agricultural Sciences, Hangzhou, China; ^3^ Joint International Research Laboratory of Agriculture and Agri-Product Safety, Yangzhou University, Yangzhou, China

**Keywords:** MITF-M, melanocytes, USP13, deubiquitination, interaction

## Abstract

Microphthalmia-associated transcription factor-M (MITF-M) is the key gene in the proliferation and differentiation of melanocytes, which undergoes an array of post-translation modifications. As shown in our previous study, deubiquitinase USP13 is directly involved in melanogenesis. However, it is still ambiguous that the effect of USP13-mediated MITF-M expression on melanocytes proliferation and apoptosis. Herein, we found that MITF-M overexpressing melanocytes showed high cell proliferation, reduced apoptosis, and increased melanin levels. Besides, melanin-related genes, TYR, DCT, GPNMB, and PMEL, were significantly up-regulated in MITF-M overexpressing melanocytes. Furthermore, Exogenous USP13 significantly upregulated the endogenous MITF-M protein level, downregulated USP13 significantly inhibited MITF-M protein levels, without altering MITF-M mRNA expression. In addition, USP13 upregulation mitigated the MITF-M degradation and significantly increased the half-life of MITF-M. Also, USP13 stabilized the exogenous MITF protein levels. In conclusion, the MITF-M level was regulated by USP13 deubiquitinase in melanocytes, affecting melanocytes proliferation and apoptosis. This study provides the theoretical basis for coat color transformation that could be useful in the development of the new breed in fur animals.

## Introduction

Melanocytes, derived from the neural crest, exert vital effects on pigmentation in mammals. Melanocyte development involving cell differentiation, proliferation, and migration is regulated by multiple biochemical and physical factors. Melanocytes are distributed in multiple mammalian tissues, such as the epidermis and dermis of the skin, hair follicle, eyes, and heart (Steingrímsson et al., 2004; Flavia et al., 2008; Yajima et al., 2010). Melanin pigments in the skin are synthesized by different melanocytes and play crucial roles in protecting mammalian skin against UV light-induced damage. Distinct distribution of pigments in mammals results in diverse coat colors (Slominski et al., 2004).

MITF-M (microphthalmia-associated transcription factor-M), a MITF isoform belonging to the MIT family, is a melanocyte-specific transcription factor with a basic-helix-loop-helix leucine zipper (bHLH-LZ) domain (Hemesath et al., 1994). It is involved in melanocyte development and functionally essential for melanocytes or melanoma (Shibahara et al., 2000; Selzer et al., 2002; Takemoto et al., 2002). Previous studies have found that as a new transcription factor, MITF-M genes mutations depleted melanocyte counts, resulting in white-colored mice (Hodgkinson et al., 1993; Hughes et al., 1994; Widlund and Fisher, 2003). Wnt signaling activates the MITF-M promoter, which is crucial for melanocyte development derived from the neural crest (Dorsky et al., 2000; Saito et al., 2003). Furthermore, MITF transcriptionally regulates crucial melanogenesis genes encoding melanin biosynthesis enzymes by mediating significant differentiation effects through a-melanocyte-stimulating hormone (α-MSH), such as TYR, TYRP1, and DCT (TYRP2) genes (Bertolotto et al., 1998; Price et al., 1998; Hartman and Czyz, 2015). In humans, MITF mutations are associated with different clinical presentations of Waardenburg Syndrome, such as deafness and hypopigmentation due to melanocytic deficiencies in the inner ear, eye, and skin (Kumawat et al., 2019; Pang et al., 2019). In melanocytes, c-Kit stimulation can regulate MITF ubiquitination, culminating in its proteolysis (Schwarz et al., 2010).

Numerous studies have shown that MITF-M is subjected to multiple post-translational modifications, such as phosphorylation and ubiquitination, affecting the activity and stability of MITF protein. The miRNA (microRNAs) are primarily involved in the post-transcriptional regulation of genes, which affects melanogenesis. MITF expression required for the formation of coat color in alpacas is subdued by the miR-25 as per microarray-based analysis (Zhu et al., 2010). In human pigmented skin, miR-218 suppressed melanogenesis through MITF repression (Guo et al., 2014). As shown previously, NO (nitric oxide) stimulation promoted MITF phosphorylation to enhance melanogenesis in alpaca skin melanocytes (Dong et al., 2011) and phosphorylation induced MITF activation promoted overexpression of *TYR*, *TYRP1*, and *TYRP2* genes, which resulted in increased melanin level (An et al., 2011).

The ubiquitin-conjugating enzyme, hUBC9, involved in melanocyte differentiation, regulates the MITF sumoylation at Lys^182^ and Lys^316^ and promotes MITF degradation (Xu et al., 2000). Ubiquitin carboxyl-terminal hydrolase L1 (UCHL1) knockdown in human melanocytes showed upregulated MITF and increased melanin content. It indicated that UCHL1 regulates skin pigmentation by reducing MITF activity via protein degradation (Seo et al., 2017). Therefore, post-translation modification plays a crucial role in regulating MITF-M activity.

Deubiquitinating enzymes (DUBs), are the distinctive proteases. DUBs can prevent target protein degradation by removing ubiquitin or ubiquitin-like proteins (Mevissen and Komander, 2017). USP13, a deubiquitinating enzyme, which belongs to the deubiquitin-specific protease family contains two highly conserved structural domains (Cys-box and His-box). It removes ubiquitin (Ub) molecules from ubiquitinated target proteins that serve as substrates (Wang et al., 2017). Previous studies have found that the deubiquitinase USP13 could reverse Mcl1 ubiquitination (Robinson et al., 2018). Previous studies have shown that deubiquitinase USP13 regulates the stability of tumor-related proteins p53 and PTEN as well as cellular antiviral response (Liu et al., 2011; Yeh et al., 2013; Zhang et al., 2013). Deubiquitinase USP13 deficiency prevented the growth of melanoma *in vitro* and *in vivo*; however, MITF overexpression prevented USP13’s inhibition, indicating MITF’s involvement in melanoma development, which was mediated by USP13 (Zhao et al., 2011). Besides, USP13 is implicated in DNA damage response, the endoplasmic reticulum stress pathway, cell cycle regulation, and innate antiviral immunity (Chen et al., 2011; Liu et al., 2014; Li et al., 2017; Sun et al., 2017).

In our previous study based on RNA-seq analysis, we showed that USP13 expression involved in melanin pigmentation was significantly different in rabbit skin with different coat colors (Qin et al., 2016). However, the role of MITF-M in melanocytes proliferation and apoptosis remains largely unexplored, and the regulatory relationship between MITF-M and USP13 remains ambiguous. Thus, it is crucial to reveal the role of MITF-M in melanocyte proliferation, apoptosis and melanogenesis, further identify the underlying molecular mechanism for MITF-M mediated regulation of USP13 in melanocytes. Therefore, MITF-M deubiquitination could unravel the molecular mechanism involved in coat color formation in mammals.

## Materials and Methods

### Ethics Statement

The experimental animal’s care and surgical procedure were approved by Jiangsu Administration Rule of Laboratory Animals and strictly followed Institutional Animal Care and Maintenance protocols. The animal experimentations were approved by the Animal Care and Use Committee at Yangzhou University (Yangzhou, China, January 15, 2021, No. 202102001).

### Experimental Animals and Skin Sample Collection

Zhejiang Yuyao Xinnong Rabbit Industry Co., Ltd provided a total of 18 adult Rex rabbits of six different coat colors (black, white, chinchilla, brown, gray, and gray yellow). Rabbits were anesthetized by injecting sodium pentobarbital into the rabbit’s ear vein. Total RNA and protein were extracted from dorsal skin samples of identical size (1 cm^2^) with the same anatomical location and subsequently, the wounds were topically treated with iodophor.

### Rabbit Melanocytes Culture

Rabbit melanocytes were cultured in Melanocyte Medium-2 (ScienCell, San Diego, CA, United States) supplemented with 1% melanocyte growth supplement-PMA-free, 0.5% fetal bovine serum (FBS), and 0.5% penicillin/streptomycin solution (ScienCell, San Diego, CA, United States), as described by Chen et al. (Chen et al., 2019).

### Tagged Vector Construction

ClonExpress II One Step cloning kit (Vazyme, Nanjing, China) was used to obtain full-length cDNA sequences of MITF-M (GenBank accession no. XM_017343949.1) and USP13 (GenBank accession no. XM_008266542.2). The MITF-M cDNA was inserted into pcDNA3.1-Flag or pcDNA3.1-Myc vectors using restriction enzymes Hind III and EcoR I. Similarly, USP13 cDNA was inserted into pcDNA3.1-Myc vectors or pcDNA3.1-Flag using restriction enzymes Hind III and Xho I. The MITF-M cloning primers are shown in Table 1.

**TABLE 1:** Primer sequences for cloning rabbit MITF-M CDS.

Primers	Sequence (5′ → 3′)
MITF-M-F	tga​acc​gtc​aga​tcc​gct​agc​ATG​CTG​GAA​ATG​CTA​GAA​TAT​AAC​CA
MITF-M-R	cga​ctg​cag​aat​tcg​aag​ctt​ACA​AGC​ATG​CTC​AGT​TTC​TTC​CA

### Cell Transfection

MITF-M and USP13 containing vectors were transfected into melanocytes using Lipofectamine™ 3,000 Reagent (Invitrogen, Carlsbad, CA, United States). Melanocytes were cultured in 24-well plates until the cell growth reached 70–90% confluency. Briefly, 1 μg plasmid diluted in 25 μl Opti-MEM™ medium (Gibco, Carlsbad, CA, United States) was added to diluted 2 μl Lipofectamine™ 3,000 (Lipofectamine™ 3,000 was diluted in 25 μl Opti-MEM™ medium). This mixture was incubated for 10–15 min at room temperature. Finally, the DNA-lipid complex was added to cultured melanocytes and maintained in an incubator at 37°C with 5% CO_2_ for 48 h, before qRT-PCR analysis.

### MITF-M siRNAs Screening

Three MITF-M siRNAs (with 5′FAM modification) and the negative control were purchased from Shanghai GenePharma Co., Ltd. (Table 2). MITF-M knockdown was performed using Lipofectamine™ 3,000 (Invitrogen, Carlsbad, CA, United States) when the melanocytes attained 70–90% confluency. 1 μl siRNA (0.264 μg/μl) and 2 μl Lipofectamine 3,000 were diluted in 25 μl Opti-MEM™ medium and it was mixed with diluted Lipofectamine 3,000 and incubated for 10–15 min at room temperature. This mixture was added to cultured melanocytes. The transfection efficiency was determined using fluorescent inverted microscopy after 12 h. The total RNA of transfected cells was extracted and analyzed using qRT-PCR after 48 h.

**TABLE 2:** Primer sequences of siRNAs.

Primers	Sequence (5′ to 3′)
Negative control	Forward: UUC​UCC​GAA​CGU​GUC​ACG​UTT
	Reverse: ACG​UGA​CAC​GUU​CGG​AGA​ATT
siRNA-MITF-M-820	Forward: GCA​GAU​GGA​UGA​UGU​AAU​UTT
	Reverse: AAU​UAC​AUC​AUC​CAU​CUG​CTT
siRNA-MITF-M-1300	Forward: GGA​GCA​CGC​UAA​CCG​GCA​UTT
	Reverse: AUG​CCG​GUU​AGC​GUG​CUC​CTT
siRNA-MITF-M-1465	Forward: GCA​GCA​UCA​UGC​AGA​CCU​ATT
	Reverse: UAG​GUC​UGC​AUG​AUG​CUG​CTT
siRNA-USP13	Forward: GGU​UAU​GGA​GGA​GCA​GCU​UTT
	Reverse: AAG​CUG​CUC​CUC​CAU​AAC​CTT

### RNA Isolation and Quantitative Real-Time PCR

Total RNA from rabbit skin and cells were extracted using RNAsimple Total RNA kit (Tiangen Biotech Co., Ltd., Beijing), as per manufacturer’s instructions. cDNA was synthesized using 1 μg total RNA and Super RT cDNA kit (Tiangen Biotech Co., Ltd.). ChamQ^TM^ SYBR^®^ qPCR Master Mix (Vazyme, Nanjing, China) was used for qRT-PCR. All qRT-PCR reactions were carried out in QuantStudio^®^5 software (Applied Biosystems; Thermo Fisher Scientific, Foster City, CA, United States). Each sample was analyzed three times and the resulting relative expression of genes was determined using the 2^−ΔΔCt^ method (Schmittgen and Livak, 2008). GAPDH was used as an internal control. The primer details of MITF-M, USP13, TYR, DCT, PMEL, and GAPDH genes are shown in Table 3.

**TABLE 3:** qRT-PCR primer sequences.

Primers	Sequence (5′ → 3′)	Product length (bp)	Application
MITF-M	Forward: GCC​TTG​GAA​CTG​GGA​CTG​AG	142	qRT-PCR
	Reverse: CCG​ACG​GCT​GCT​TGT​TTT​AG		
USP13	Forward: TCG​CCA​TAC​AGA​AAG​CAG​G	165	—
	Reverse: ATC​GGT​CAG​ATT​CAA​CCA​GAG		
TYR	Forward: CTC​TTC​TTG​TTG​CTG​TGG​G	156	—
	Reverse: GCT​GAG​TAG​GTT​AGG​GTT​TTC		
DCT (TYRP2)	Forward: ATT​CTG​CTG​CCA​ATG​ACC​C	154	—
	Reverse: AAC​GGC​ACC​ATG​TTA​TAC​CTG		
PMEL	Forward: GTC​AGC​ACC​CAG​CTT​GTC​A	130	—
	Reverse: GCT​TCA​TTA​GTC​TGC​GCC​TGT		
GPNMB	Forward: TCC​AGA​TTG​CAG​AAG​TCC​CGA​T	173	—
	Reverse: GCA​GCT​CTC​AGT​CTC​GTC​CA		
GAPDH	Forward: CAC​CAG​GGC​TGC​TTT​TAA​CTC​T	141	—
	Reverse: CTT​CCC​GTT​CTC​AGC​CTT​GAC​C		

### Protein Preparation and Wes Simple Western Analysis

Total protein from rabbit skin was isolated using cell lysis buffer with 1% phenylmethanesulfonyl fluoride (PMSF) (Beyotime, Shanghai, China) for Wes simple western analysis. The protein concentration of the lysed cells was estimated using the BCA protein assay kit (Beyotime, Shanghai, China). Protein assay was performed using Wes̛s automated western blotting system (ProteinSimple) (Harris, 2015). Anti-GAPDH mouse monoclonal antibody (Abcam, Cambridge, United Kingdom), anti-MITF mouse monoclonal antibody (SANTA CRUZ, Dallas, TX, United States), USP13 monoclonal antibody (Proteintech, Wuhan, China) in 1: 100 dilution was used.

### Melanin Content Measurement

Transfected melanocytes post 72 h were washed using phosphate-buffered saline (PBS) (HyClone, Logan, United States) three times lightly. Later, these melanocytes were centrifuged and the supernatant was discarded. The pelleted cells were lysed using 1 M NaOH and incubated at 80°C for 1 h. Finally, optical density (OD) was measured at 475 nm using an Infinite M200 PRO (Tecan, Männedorf, Switzerland) spectrophotometer.

### Cell Proliferation Assay

12 h post-transfection, transfected cells were seeded into 96-well plates using Cell Counting Kit-8 assay (Vazyme, Nanjing, China), as per the manufacturer’s instructions. Later, OD at 450 nm was measured at 0, 24, 48, and 72 h using an Infinite M200 Pro spectrophotometer (Tecan, Männedorf, Switzerland).

### Apoptosis Assay

36 h post-transfection, transfected cells were harvested. Apoptosis assay was performed using an Annexin V-FITC apoptosis detection kit (Vazyme, Nanjing, China). Later, the cells were sorted using a fluorescence-activated cell sorter (FACSAria SORP flow cytometer, Becton Dickinson, San Jose, CA, United States). OD of each sample was measured three times.

### Co-Immunoprecipitation

Melanocytes were cotransfected with pcDNA3.1-Myc-MITF-M and pcDNA3.1-Flag-USP13 plasmids using Lipofectamine™ 3,000 Reagent (Invitrogen, Carlsbad, CA, United States). After 72 h, transfected melanocytes were collected and lysed for 1 h using 1 ml RIPA Lysis Buffer at 4°C. These cells were centrifuged at 12, 000 g for 10 min and the supernatant was separated. Cell lysates were incubated with Protein A/G agarose and IgG as the control for 2 h to remove non-specific binding proteins. Later, cell lysates were centrifuged to remove Protein A/G agarose, and the supernatant (80 μl) was collected for western blot assay. The rest of the lysates were incubated with DYKDDDDK Tag Monoclonal Antibody (Binds To FLAG^®^ Tag Epitope) (Proteintech, Wuhan, China) overnight at 4°C. Then, Protein A agarose beads that were washed using RIPA Lysis Buffer were added to the lysates containing DYKDDDDK Tag Monoclonal Antibody and incubated for 2 h at 4°C. Finally, cell lysates were centrifuged, and the supernatant was collected for immunoblot analysis after immunoprecipitation.

### Deubiquitination Assay

MITF-M plasmids containing Flag tag and USP13 plasmids containing Myc tag were cotransfected in melanocytes. Cell lysates were collected and immunoprecipitated using anti-Flag M2 magnetic bead at 4°C overnight with gentle shaking. Immunoprecipitated complexes were adsorbed using magnetic stand and the supernatant was discarded, the rest precipitates were washed and resuspended using IP Buffer for four times. Finally, the lysates were discarded as much as possible, and 5 × SDS-PAGE loading buffer was added to the rest immunoprecipitates, at 100°C for 5 min. Later, the supernatant was collected for immunoblot analysis.

### Statistical Analysis

All data were analyzed using SPSS version 25 (SPSS Inc., Chicago, IL, United States). One-way ANOVA was employed to analyze significant differences. All values represent means ± standard deviation (SD).

## Results

### MITF-M is Involved in the Formation of Different Coat Colors in Rex Rabbits

**FIGURE 1:**
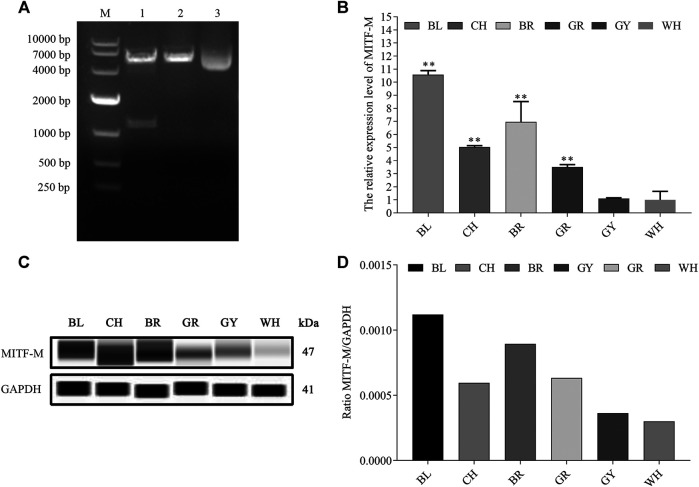
The mRNA and protein expression levels of MITF-M in rabbit skin with different coat colors. **(A)** The rabbit MITF-M cDNA was successfully cloned and reconstructed into pcDNA3.1-Flag vectors. M, DL10000 DNA Marker. Lane 1, pcDNA3.1(+)-Flag-MITF-M plasmid was digested by restriction enzymes *Hind* III and EcoR I. Lane 2, pcDNA3.1(+)-Flag-MITF-M plasmid was digested by restriction enzymes Hind III. Lane 3, pcDNA3.1(+)-Flag-MITF-M plasmid was not digested. **(B)** The MITF-M mRNA level in rabbit skin with different coat colors was determined through qRT-PCR. Each sample had three replicates, and the relative expression levels of genes were determined using GAPDH as an internal control by using the 2^−ΔΔCt^ method. The significant differences among the studied groups were analyzed by using the white rabbit’ skin as the control group, the fold change of other studied groups was calculated by the expression level of the white rabbit’ skin was normalized to 1. Each group is an independent group. Least—Significant Difference (LSD) was employed to compare the studied groups to the control group. ^**^ indicates *p* < 0.01. **(C)** The MITF-M protein expression in rabbit skin with different coat colors. **(D)** MITF-M protein differential expression trend in Rex rabbits with different coat colors. The relative expression ratio of MITF-M/GAPDH was quantified by the area of Wes. BL, black; CH, chinchilla; BR, brown; GR, gray; GY, gray-yellow; WH, white.

Based on the mammalian MITF-M sequence, the amino-terminal with unique M domain (MLEMLEYNHY) as well as specific primers were designed. Rabbit MITF-M cDNA was successfully cloned according to the reference sequence in NCBI (GenBank accession no. XM_017343949.1), containing 1,242 bp coding sequence (CDS) (Figure 1A). As shown in our previous study, MITF-M is involved in the coat color formation of Rex rabbits. The MITF-M expression levels were detected in rabbit’s skin with different coat colors using qRT-PCR and Wes assays. As per the outcomes, the highest and lowest change in mRNA and protein expression levels of MITF-M was observed in black coat colored rabbit skin and white coat colored rabbit skin, respectively (*p* < 0.01) (Figures 1B–D).

### MITF-M Regulated the Expression of Melanin-Related Genes in Melanocytes

**FIGURE 2:**
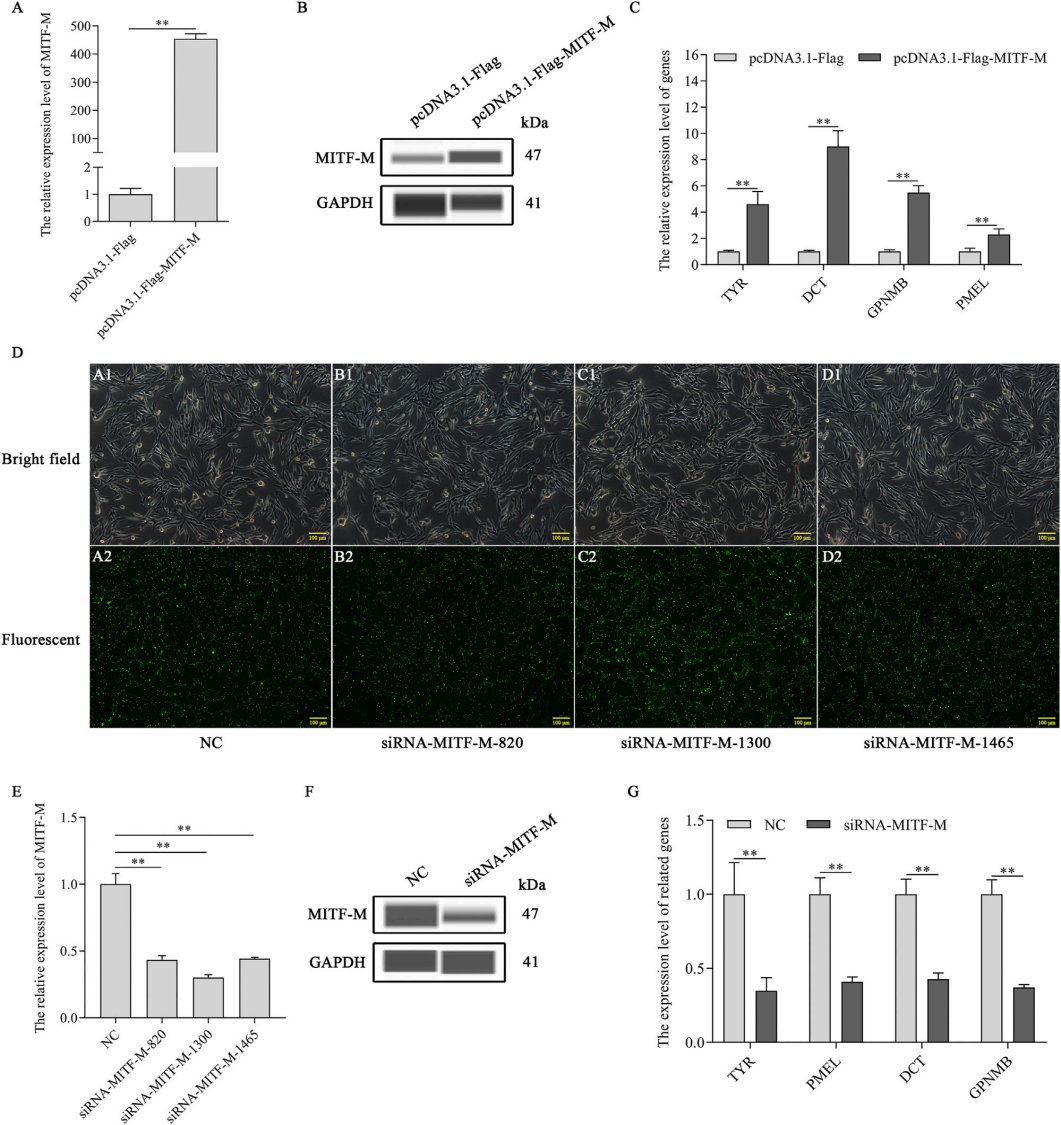
MITF-M regulated the expression of the downstream melanin-related genes. **(A)** MITF-M mRNA expression level, as per the qRT-PCR based analysis, in MITF-M overexpressing melanocytes. **(B)** The MITF-M protein expression was determined by Wes in MITF-M overexpressing melanocytes. **(C)** The qRT-PCR assay was performed to detect the mRNA expression levels of melanin-related genes in MITF-M overexpressing melanocytes. **(D)** The MITF-M siRNAs containing 5′FAM modification were transfected in melanocytes to determine its transfection efficiency, and transfected cells were observed using fluorescent inverted microscopy (100×). **(E)** The MITF-M mRNA expression was determined in MITF-M silenced melanocytes using qRT-PCR. **(F)** The MITF-M protein expression was determined in MITF-M silenced melanocytes using Wes. **(G)** The expression of melanin-related genes was determined after MITF-M downregulation in melanocytes using qRT-PCR. Each sample had three replicates, and the relative expression of genes was determined using GAPDH as an internal control by using the 2^−ΔΔCt^ method. ^**^ indicates *p* < 0.01.

As a transcription factor, MITF-M plays a crucial role in regulating the expression of the downstream genes. The mRNA expression levels of melanin-related genes were determined post-MITF-M overexpression and knockdown in melanocytes. As per the outcomes, *MITF-M* mRNA and MITF-M protein levels (Figures 2A,B) and that of downstream melanin-related genes, TYR, DCT, GPNMB, and PMEL, were upregulated significantly in MITF-M overexpressing melanocytes (*p* < 0.01) (Figure 2C). Furthermore, melanocytes were transfected with MITF-M siRNAs containing 5’ FAM modification. As per the qRT-PCR based analysis, three siRNAs showed high transfection efficiency (Figure 2D), and the siRNA-1,300 showed the highest interference efficiency, which was significantly lower than the negative control (*p* < 0.01) (Figure 2E). MITF-M knockdown using siRNA-1,300 decreased the MITF-M protein level (Figure 2F). mRNA expression levels of melanin-related genes also decreased significantly (*p* < 0.01) (Figure 2G).

### MITF-M Increased Melanin Synthesis by Promoting Proliferation and Inhibiting Apoptosis in Melanocytes

**FIGURE 3:**
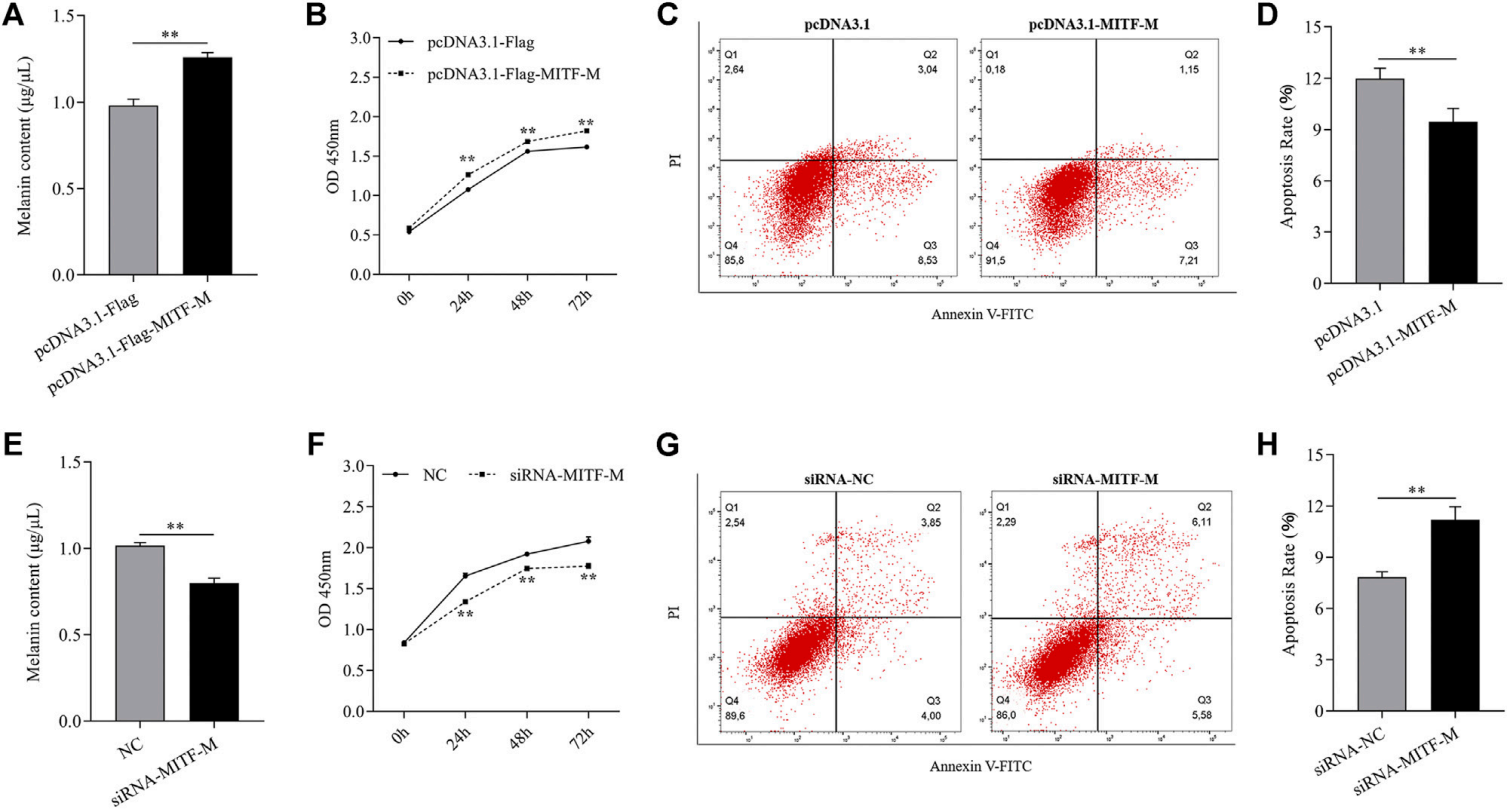
MITF-M regulated melanin synthesis, proliferation, and apoptosis of melanocytes. **(A)** The melanin content in MITF-M overexpressing melanocytes. **(B)** Melanocyte proliferation was estimated using CCK-8 assay at 24, 48, and 72 h in MITF-M overexpressing melanocytes. **(C)** Melanocyte apoptosis in MITF-M overexpressing melanocytes. **(D)** Cellular apoptosis rate in MITF-M overexpressing melanocytes. **(E)** The melanin content in MITF-M knockdown melanocytes. **(F)** Melanocyte proliferation was estimated in MITF-M knockdown melanocytes at 24, 48, and 72 h using CCK-8 assay **(G)** Cellular apoptosis in MITF-M inhibited melanocytes. **(H)** The cellular apoptosis rate in melanocytes was calculated post-MITF-M interference. Each sample had three replicates. ^**^ indicates *P* < 0.01.

To discern the effects of MITF-M on melanocyte development, the melanin content was measured in MITF-M overexpressing or knockdown melanocytes. MITF-M overexpressing melanocytes showed a significant increase in melanin content (*p* < 0.01) (Figure 3A) and cell proliferation (*p* < 0.01) (Figure 3B) and a significant decrease in apoptosis rate (*p* < 0.01) (Figures 3C,D). Subsequently, melanin content (*p* < 0.01) (Figure 3E) and cell proliferation (*p* < 0.01) (Figure 3F) was significantly decreased in melanocytes with MITF-M knockdown; however, apoptosis was significantly increased (*p* < 0.01) (Figures 3G,H).

### Endogenous MITF-M Protein Levels Were Regulated by USP13 in Melanocytes

**FIGURE 4:**
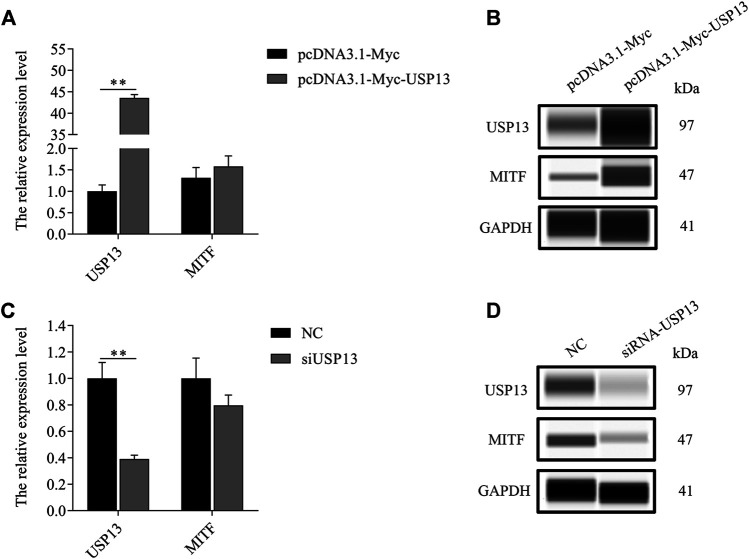
Exogenous USP13 regulated endogenous MITF protein. **(A)** *MITF-M* mRNA expression was determined in USP13 overexpressing cells. **(B)** Endogenous MITF protein was detected in USP13 overexpressing cells. **(C)** The MITF-M mRNA expression in USP13 knockdown cells treated with siRNA. **(D)** Endogenous MITF protein was detected post USP13 overexpression. Each sample had three replicates. ^**^ indicates *p* < 0.01.

As per the outcomes of our analysis, MITF-M plays a crucial role in the melanogenesis of melanocytes. However, deubiquitinase USP13 mediated regulation of MITF-M expression remains obscure. Thus, MITF-M expression in USP13 overexpressing or knockdown melanocytes was analyzed. MITF-M protein levels were substantially increased in USP13 overexpressing, and significantly reduced in USP13 downregulated cells treated with siRNA, without altering MITF-M mRNA expression (Figure 4). Thus, we observed that USP13 could regulate MITF-M at the level of a post-translational modification.

### USP13 Interacted With MITF-M and Regulated MITF-M Protein Stability

**FIGURE 5:**
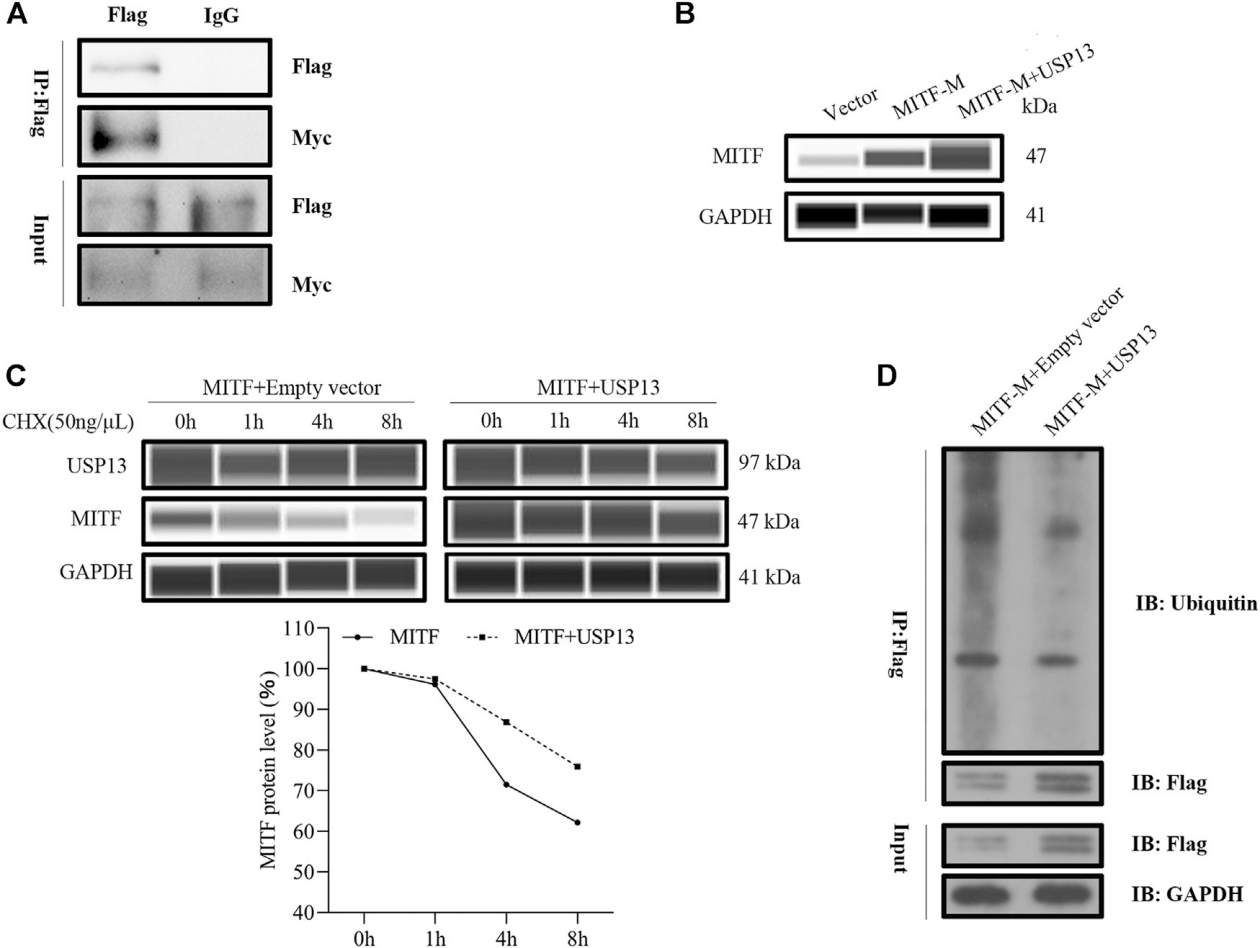
Exogenous USP13 interacted with MITF-M and stabilized the exogenous MITF protein levels. **(A)** Melanocytes co-transfected with Flag-tagged USP13 and Myc-tagged MITF-M were immunoprecipitated using anti-Flag antibody and the protein complex was detected through western blot analysis using anti-Myc and anti-Flag antibodies. **(B)** MITF-M protein was detected in MITF-M overexpressing group, and pcDNA3.1-Myc-USP13 and pcDNA3.1-Flag-MITF-M co-transfected melanocytes using Wes. **(C)** Melanocytes were transfected with MITF-M, USP13, or plain vector and incubated for 3 days. Later, the transfected cells were treated with 50 ng/μl CHX for 1, 4, and 8 h. Cells were collected at the indicated time points, and MITF, USP13, and GAPDH protein levels were measured by Wes using MITF, USP13, and GAPDH antibodies. The MITF protein bands were quantified by calculating the area of Wes and normalized using GAPDH. **(D)** MITF-M ubiquitination level was determined after pcDNA3.1-Flag-MITF-M and pcDNA3.1-Myc-USP13 were co-transfected in melanocytes. The anti-Flag antibody was used to immunoprecipitate, the anti-GAPDH mouse monoclonal antibody and anti-Flag antibody were used in western blot assay.

To explore the mechanism of USP13 regulated changes in MITF-M protein levels, USP13 and MITF-M were overexpressed in melanocytes, and interaction between exogenous MITF-M and exogenous USP13 was detected in these cells using Co-IP assay (Figure 5A). Furthermore, MITF-M protein was overexpressed post USP13 and MITF-M, co-transfection as compared to cells transfected with only MITF-M (Figure 5B). To investigate the USP13 regulated MITF-M protein stability, MITF-M and USP13 containing vectors were co-expressed in melanocytes. 72 h post-transfection, these cells were treated with cycloheximide (CHX) at specific time points. As per the outcomes, USP13 upregulation mitigated the MITF-M degradation and significantly increased the half-life of MITF-M (Figure 5C). It indicated that USP13 stabilized the exogenous MITF protein levels.

To further verify whether MITF-M protein ubiquitination level was affected by deubiquitinase USP13, MITF-M and USP13 plasmid were cotransfected in melanocytes, a ubiquitination assay that the change of MITF-M ubiquitination level was performed. MITF-M ubiquitination level was determined using ubiquitin antibody. The outcomes of this analysis demonstrated that MITF-M ubiquitination level was apparently inhibited by USP13 deubiquitination, compared to the control group, which conained melanocytes transfected with pcDNA3.1-Flag-MITF-M and pcDNA3.1-Myc plasmids (Figure 5D).

## Discussion

Different coat colors in rabbits are due to different melanin levels, which are regulated by several essential genes in melanocytes. In this study, we observed that MITF-M mRNA and MITF-M protein levels were not the same in the rabbit skin with different coat colors. The highest expression of MITF-M was observed in the black coat colored rabbit skin, and the white coat colored rabbit skin showed the lowest expression of MITF-M. As shown in a previous study, MITF-M mRNA and MITF-M protein levels were the lowest in rabbit skin with black coat color and the highest in mice skin with brown color post-miR-137 overexpression, and it negatively affected the coat colors, and down-regulated MITF protein (Dong et al., 2012; Chen et al., 2018). As reported previously, MITF-M has a unique promoter, known as the M promoter, which is explicitly activated in melanocytes (Fuse et al., 1996). It is speculated that MITF-M affects pigmentation in other species as well. For instance, the mutation in the M promoter gives rise to MITF major melanocyte isoform resulting in white spots in horses and dogs (Hauswirth et al., 2012; Baranowska Körberg et al., 2014). Chicken skin color is altered based on the mutation of MITF promoter in black-boned chicken (Wang et al., 2018).

MITF-M, the master regulator of melanocyte differentiation, is a crucial transcription factor that regulates melanin-related gene expression. It has been reported that melanin-related genes, such as TYR and TYRP1, play a crucial role in melanin biosynthesis (Hartman and Czyz, 2015). Tyrosinase is a rate-limiting enzyme in melanin synthesis, which can determine the eumelanin/pheomelanin ratio (Wagner et al., 2001). The pheomelanin level increased when tyrosinase content decreased (Ito, 2003). GPNMB, a distinctive melanosomal protein, affects the transcriptional regulation of melanocytes. Previous studies demonstrated that the GPNMB expression was identical to that of MITF and DCT, which was remarkably reduced in MITF mutated melanocytes (Loftus et al., 2009). As per the outcomes of our analysis, TYR, DCT, GPNMB, and PMEL expression were regulated by MITF-M. The expression of melanin-related genes was significantly increased in MITF-M overexpressing melanocytes. Conversely, the mRNA levels of these genes decreased apparently in MITF-M silenced cells using siRNA.

Melanocytes are well-differentiated and melanin-producing cells. Melanocytes of different embryonic origins synthesize distinct melanin types, which have a unique function at different target locations (Plonka et al., 2009). Proliferation and apoptosis are crucial components of melanocyte development. It is a well-known fact that cell proliferation is inhibited during cell differentiation. It was speculated that epidermal melanocytes do not proliferate under normal circumstances (Cichorek et al., 2013). MITF functions as a crucial adaptor to regulate melanocyte proliferation, apoptosis, and differentiation (Becker et al., 2009; Margue et al., 2013; Nicholas et al., 2013; Xu et al., 2013). Overexpression of BCL2, an anti-apoptotic gene, mitigates apoptosis by MITF disruption in melanocytes (McGill et al., 2002). The MET promoter activation mediated by MITF prevents apoptosis in melanocytes (Beuret et al., 2007). As per the outcome of our analysis, melanin content was increased in MITF-M overexpressing melanocytes. MITF-M upregulation promoted cell proliferation and inhibited apoptosis in melanocytes. Conversely, apoptosis increased in siRNA*-*treated MITF-M knockdown cells, decreasing cellular proliferation. In conclusion, our results indicated that MITF-M, as a transcription factor, promotes melanocyte development.

Protein ubiquitination is a post-translational protein modification, which participates in multiple biological processes, such as DNA repair, signal transduction, and melanocyte proliferation (Di Fiore et al., 2003; Feng et al., 2015; Yatsu et al., 2015). As per previous studies, ubiquitination promotes Melan-A/MART-1 degradation in melanosomes present in melanocytes (Lévy et al., 2005). To inhibit melanocyte proliferation, E3 ligase APC/C(Cdh1) enhanced ubiquitination and degradation of the transcription factor PAX3 (Cao et al., 2015). Thus, we speculated that deubiquitination has a vital effect on melanocyte development. As per the current study, MITF-M post-translational levels were regulated by deubiquitinase USP13. MITF-M protein level was significantly reduced when the USP13 expression was suppressed by siRNA. Conversely, USP13 upregulation promoted MITF-M protein levels, without influencing MITF-M mRNA levels. MITF-M ubiquitination level was reduced by USP13 deubiquitinase. Furthermore, MITF-M stability was augmented by USP13. It demonstrated that USP13 regulates melanocyte expression by modulating MITF-M stability.

In summary, MITF-M acts as the master regulator of melanogenesis, melanocyte proliferation, and apoptosis. USP13 deubiquitination positively affects MITF-M stability in melanocyte development. USP13 is a crucial deubiquitinase, which regulates melanocyte development and determines coat colors in mammals.

## Data Availability

The datasets presented in this study can be found in online repositories. The names of the repository/repositories and accession number(s) can be found below: The following information was supplied regarding raw data availability: https://doi.org/10.6084/m9.figshare.14169488.v1.

## Abbreviations

CHX, Cycloheximide; DCT (TYRP2), Tyrosinase related protein 2; DUBs, Deubiquitinating enzymes; FBS, Fetal bovine serum; GAPDH, Glyceraldehyde-3-phosphate dehydrogenase; GPNMB, Glycoprotein non-metastatic melanoma protein B; IP, Immunoprecipitation; MITF-M, Microphthalmia-associated transcription factor-M; NC, Negative control; OD, Optical density; PBS, Phosphate buffer saline; PMEL, Premelanosome protein; qRT-PCR, quantitative real time PCR; RIPA, Radio-immunoprecipitation assay; SD, Standard deviation; TYR, Tyrosinase
